# Otology

**Published:** 1905-05-06

**Authors:** 


### Progress in Medicine and Surgery.

### OTOLOGY.

(*Continued from page* 91.)

**Otitic Cerebellar Abscess.**—H. Betham Robinson[4] reports a successful operation for a case of this kind in a boy of 13. There had been left ear discharge for eight years, but this had stopped three weeks before the operation, and for this period there had been incessant vomiting, obstinate constipation, rapid loss of flesh, pain in the head, and drowsiness. There was slight œdema and tenderness in the mastoid region. The pupils were equal, the optic discs blurred. The grip of the left hand, the side of the disease, was weaker than that of the right. The patient tended to lie curled up on his right side. The temperature was 104° F. on admission to hospital. A radical mastoid operation was performed. Pus appeared to come from the posterior part of the antrum. The sigmoid sinus was then exposed and slightly lacerated. It bled freely through the laceration showing absence of obstructive thrombosis. The pus did not come from the sigmoid groove but through the posterior wall of the antrum, just in front of the sinus. It was very offensive. The pus cavity was drained with a silver drainage tube. A rigor occurred on the following day and it was deemed advisable to tie the jugular vein. Complete recovery followed gradually in six weeks. Mr. Robinson discusses the symptoms. At St. Thomas's Hospital cerebellar otitic abscess has been met with far oftener than temporo-sphenoidal abscess. The symptoms pointing to cerebellar abscess were the weakness of the upper limb, the increase of the knee-jerk on the side of the disease (left), and the tendency of the patient to curl himself up on the opposite side. These symptoms have resulted from the experimental removal of one lateral lobe of the cerebellum as demonstrated by Luciani and Risien Russell, and confirmed clinically by Acland and Ballance. Luciani believes that the weakness of the limbs on the side of the lesion is due to the loss of a reinforcing influence of the lateral lobe of the cerebellum upon the opposite cerebral hemisphere. The fibres involved would be those passing through the superior cerebellar peduncles. These fibres are particularly related to the dentate nucleus, some passing through the nucleus to the portion of the cerebellar cortex deeply placed on its inner and front part. The abscess in the above case was situated in this part of the cerebellum. Conjugate deviation of the eyes to the opposite side, observed in some cases, was not present. Nystagmus was not noticed until the second day after the operation, and the movements were away from the lesion. They should be towards the affected side. Perhaps in this case they were the result of injury to the horizontal semicircular canal. When the rigor followed the operation it was thought that it might be due to infection through the laceration of the lateral sinus which had been made at the operation, and for this reason the jugular vein was ligatured. It was difficult to say whether the opening in the posterior antral wall in front of the sinus had just been formed or had existed previously. It is well known that cerebral and cerebellar abscesses may discharge spontaneously through the middle ear. The fact that such an opening may be found on opening up the antrum and tympanum shows the desirability of carrying out this procedure first when the existence of such otitic abscesses is suspected. MacEwen successfully dealt with an abscess in the same way as the author.

[4] Lancet, Dec. 3, 1904, p. 1566.

